# Association between a Single Nucleotide Polymorphism in the 3′-UTR of *ARHGEF18* and the Risk of Nonidiopathic Pulmonary Arterial Hypertension in Chinese Population

**DOI:** 10.1155/2018/2461845

**Published:** 2018-10-14

**Authors:** Ding Li, Yan Sun, Xiaochao Kong, Changxing Luan, Youjia Yu, Feng Chen, Peng Chen

**Affiliations:** ^1^Department of Forensic Medicine, Nanjing Medical University, Nanjing, Jiangsu 211166, China; ^2^Department of Oncology, Jiangsu Cancer Hospital, Nanjing Medical University, Nanjing, Jiangsu 211166, China; ^3^Forensic Expertise Institute, Nanjing Medical University, Nanjing, Jiangsu 211166, China

## Abstract

*ARHGEF18* has been identified as upregulated in the lung tissues of rat models of pulmonary artery hypertension introduced by hypoxia or monocrotaline (MCT). We used online SNP function prediction tools to screen the candidate SNPs that might be associated with the regulation of the ARHGEF18 expression. The result suggested that rs3745357 located in the 3′-untranslated region of *ARHGEF18* is probably a genetic modifier in the process. In the present study, we aimed to investigate the association between *ARHGEF18* rs3745357 polymorphism and nonidiopathic pulmonary arterial hypertension susceptibility (niPAH). A total of 293 participants were included in the case-control study (117 patients and 176 healthy controls). The rs3745357 variant was discriminated by using cleaved amplification polymorphism (CAP) sequence-tagged site technology. Although the overall allele and genotype frequencies of rs3745357 in niPAH patients were close to those of the control group, significant differences have been identified when we further divided the niPAH patients into subgroups with or without coronary heart disease (CHD). Rs3745357 C allele frequency was significantly higher in niPAH patients without CHD history (*p* = 0.001), while the frequency was significantly lower in niPAH patients with CHD history (*p* = 0.017) when compared to control subjects. The distribution of genotype frequencies was also quite different. After adjustment by gender and age, significant differences were found between patients with CHD history and controls. The results suggest that the ARHGEF18 rs3745357 variant may be used as a marker for the genetic susceptibility to niPAH.

## 1. Introduction

Pulmonary arterial hypertension is a progressive and lethal disease which occurs at the small pulmonary arteries and is characterized by increased pulmonary vascular resistance due to vascular proliferation and remodeling [1]. Although growing studies revealed that multiple mechanisms contributed to disease development [2], the exact pathogenesis of PAH remains unclear. However, several experimental models have been used to understand the mechanism underlying the pathogenesis. The most commonly used model is the monocrotaline (MCT) rat model. In this model, MCT is injected subcutaneously and becomes metabolically activated, as a pyrrolizidine alkaloid, by hepatic cytochrome P450 3A [3, 4]. The active MCT pyrrole is pneumotoxic and damages the pulmonary artery endothelial cells (PAECs), which leads to a disturbed barrier function [5]. MCT could lead to pulmonary vascular remodeling by inducing arterial medial hyperplasia of the axial arteries, interstitial oedema, adventitial inflammation, haemorrhage, and fibrosis [6, 7]. Eventually, pulmonary vascular resistance increases and the right ventricle compensates by hypertrophy [8, 9]. In addition to the MCT PAH rat model, hypoxia is also widely used to study experimental PAH [10]. MCT- or hypoxia-treated animals can be induced to develop PAH suggesting that the expression level of certain genes could be altered. These altered genes may be involved in the development of PAH.

PAH and coronary heart disease (CHD) are circulatory system diseases that may simultaneously emerge in a patient and they are often treated together in clinical practices. CHD is common among patients with PAH [11]. A previous study has identified 29 overlapping genes between the two diseases which demonstrated that genetic similarity existed [12]. However, the occurrence and impact of CHD in patients with PAH are still unknown.

In the year 2000, ARHGEF18 (p114RhoGEF), a novel guanine nucleotide exchange factor (GEF) for Rho GTPases which is widely expressed in human tissues, was identified [13]. ARHGEF18 regulates the activity of RhoA and Rac1, and G*βγ* subunits of heterotrimeric G proteins are activators of ARHGEF18. The results demonstrated the role played by ARHGEF18 in actin stress fiber formation, cell shape change, and reactive oxygen species (ROS) production [14]. ARHGEF18 is a component of a junction-associated Rho signaling module that drives spatially restricted activation of RhoA to regulate junction formation and epithelial morphogenesis [15]. ARHGEF18 controls RhoA activity in the human bronchial epithelial cell line 16HBE14o- (16HBE) to promote apical junction assembly. The abnormal *ARHGEF18* expression level and DNA variants in the gene have been associated with different diseases, including adult-onset retinal degeneration, systemic capillary leak syndrome, and squamous-cell lung carcinoma [16–18].

In the present study, 293 participants were recruited, including 117 niPAH patients and 176 healthy control subjects. A genetic epidemiological study is cost-effective for exploring the association between genetic variation and diseases. This study is aimed at investigating the influence of *ARHGEF18* polymorphism on the prevalence of niPAH.

## 2. Materials and Methods

### 2.1. Subjects

The present study was composed of 117 cases of niPAH and 176 controls. Patients were consecutively recruited from the Affiliated Hospital of Jiangsu University between May 2014 and July 2017. Clinical information was obtained from pathological records, including gender, age, drinking habits, smoking, and CHD history. Baseline profiles of the study populations are summarized in Table 1. The control subjects were collected from healthy volunteers who visited the Sir Run Run Hospital Nanjing Medical University for medical examination during the same period. Informed consent was obtained from each participant.

### 2.2. SNP Selection

The SNPinfo web tool was employed to screen the potential functional SNPs within the *ARHGEF18* gene. We mainly focused on the 3′-UTR region, which could be a potential functional region. Among the 10 potential functional SNPs, rs3745357 and rs1043412 have a minor allele frequency > 0.1 in the Han Chinese population. The two SNPs are also in complete linkage disequilibrium. To the best of our knowledge, no studies have examined the role of rs3745357 or rs1043412 polymorphisms within *ARHGEF18* in the development of PAH so far.

### 2.3. Genotyping

**Figure 1:**
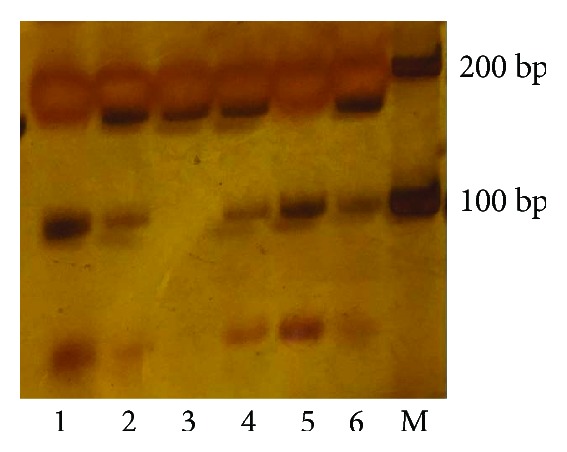
Lanes 1 and 5 are CC genotypes; lanes 2, 4, and 6 are CT genotypes; lane 3 is a TT genotype; and M is the DNA ladder from 100 to 600 bp.

Genomic DNA was extracted from 200 *μ*l of EDTA-anticoagulated peripheral blood using a commercial extraction kit (Tiangen Biotech Corporation, Beijing, China) according to the instruction manual. We performed a polymerase chain reaction-restriction fragment length polymorphism assay to detect the genotype of the SNP. Primer sequence was forward: 5′-AGAGAACATTCCCAGACCCTG-3′ and reverse: 5′-AGACCCCTCTGCCACATG-3′. The restriction enzyme used was Hpy188I, and its C allele is cuttable, yielding two fragments of 98 bp and 59 bp (Figure 1). About 10% of the samples were randomly selected to perform repeated assays and acquire the results.

### 2.4. Statistical Analysis

All data were analyzed using SPSS 13 (SPSS Inc., Chicago, IL). Genotype frequencies of the SNP were obtained by directed computing. Genotypic association analysis in a case-control pattern assuming codominant, dominant, recessive, and overdominant genetic models was performed using SNPstats. Odds ratio (OR) and respective 95% confidence intervals were reported to evaluate the effects of any differences between allele and genotype frequencies. A probability of 0.05 or less was regarded as statistically significant in patients with PAH compared to healthy controls.

## 3. Results

**Figure 2:**
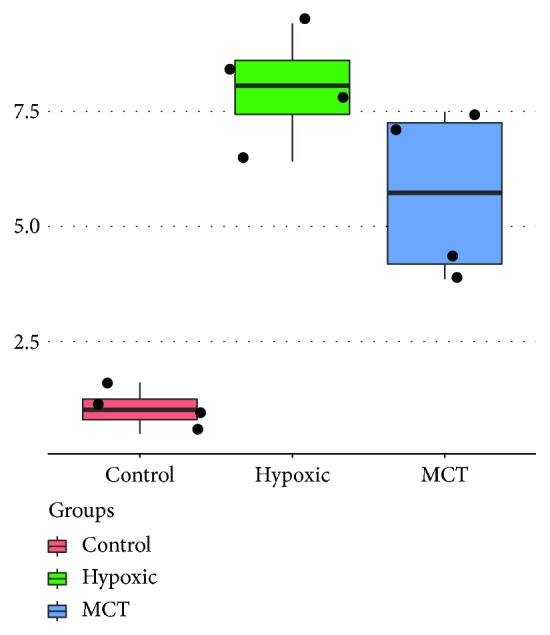
Expression values of ARHGEF18 in rats under different processing methods. Data from GEO datasets for GSE72707 (https://www.ncbi.nlm.nih.gov/geo/query/acc.cgi?acc=GSE72707).

To confirm the expression level of *ARHGEF18*, we screened the expression profiling result published on the GEO database, which was produced by using Agilent-028282 Whole Rat Genome Microarray (https://www.ncbi.nlm.nih.gov/geo/query/acc.cgi?acc=GSE72707). The dataset profiled the lungs of adult male Sprague-Dawley rats kept for 4 weeks in normal atmospheric conditions or in hypobaric hypoxia (380 mmHg), or injected with 40 mg/kg MCT. The *ARHGEF18* expression value was increased both when treated with hypoxia (logFC = 6.958, *p* = 2.96 × 10^−6^) or MCT (logFC = 4.67, *p* = 1.59 × 10^−4^), respectively (Figure 2).

The *ARHGEF18* 3′-UTR variant rs3745357 was successfully genotyped in 117 niPAH patients and 176 healthy controls. When the niPAH patients were divided as those with or without the history of CHD, significant differences were observed between the patients and controls. In niPAH patients without CHD history, the frequency of C allele carriers was significantly higher than the controls. Conversely, in the niPAH patients with CHD history, the frequency of C allele carriers was significantly lower than the controls (Table 2). As shown in Table 3, a significantly increased niPAH risk was associated with the CC genotype (OR = 3.94, 95% CI = 1.58–9.80, *p* = 0.0053) when compared to the TT genotype. In the dominant model, CT/CC genotype carriers had a 2.34-fold decreased niPAH susceptibility compared to TT genotype carriers. In the recessive model, CC genotype carriers also had a 2.93-fold increased risk to develop PAH compared to TT/CT genotype carriers.

Table 4 lists the differences between the PAH patients with CHD history and controls. In contrast to the patients without CHD history, CT or CT/CC genotype carriers were observed having significantly decreased niPAH risk when compared to TT or TT/CC genotypes in codominant (CT versus TT: OR = 0.38, 95% CI = 0.20–0.72, *p* = 0.0092), dominant (CT/CC versus TT: OR = 0.43, 95% CI = 0.24–0.75, *p* = 0.0031), and overdominant (CT versus TT/CC: OR = 0.47, 95% CI = 0.26–0.85, *p* = 0.011) models, respectively. When we further adjusted the results by sex and age, significant differences were also observed between patients and controls in codominant (CC versus TT: OR = 0.03, 95% CI = 0.00–0.46, *p* = 0.011), dominant (CT/CC versus TT: OR = 0.10, 95% CI = 0.02–0.69, *p* = 0.009), and recessive (CC versus TT/CT: OR = 0.11, 95% CI = 0.01–0.93, *p* = 0.031) models, respectively.

## 4. Discussion

*ARHGEF18* encodes ARHGEF18 (also known as p114RhoGEF), the Rho/Rac guanine nucleotide exchange factor 18. The gene is essential for podocyte cytoskeletons and plays diverse roles in regulating collective cell migration [19, 20]. Kim et al. has reported that ARHGEF18 governs cell motility and lumen formation during tubulogenesis through a ROCK-myosin-II pathway [21]. Other studies revealed that ARHGEF18 could regulate the Rho signaling pathway [15, 22–25]. Abnormal *ARHGEF18* expression levels and DNA variants in the gene have been associated with different diseases, including adult-onset retinal degeneration, systemic capillary leak syndrome, and squamous-cell lung carcinoma [16–18]. Evidence that the Rho kinase pathway contributes to vasoconstriction in PAH is demonstrated by the effects of Rho kinase inhibitors to acutely reduce PAH in both chronically hypoxic rats and neonatal rats with PAH [26–29]. Rho kinase-mediated Ca^2+^ sensitization also plays an important role in mediating enhanced basal pulmonary arterial tone as well as agonist- and depolarization-dependent vasoconstriction in small pulmonary arteries from animal models.

Previous studies identified numerous biological characteristics of PAH and CHD. There were 29 overlapping genes with genetic similarity between PAH and CHD. The existing data strongly suggested that CHD might have an impact on the patients with PAH. Rs3745357 is located in the 3′-UTR region of *ARHGEF18*. In many genes, the 3′-UTR plays an important role in regulating transcription and protein expression. SNPs in the 3′-UTR region may be able to influence either of these processes in protein expression. In the present study, we have provided primary evidence that the C allele of the rs3745357 polymorphism is more frequent in PAH patients without CHD history, while it is less frequent in PAH patients with CHD history, compared with control subjects. Moreover, the frequencies of the CT/CC genotypes of the rs3745357 polymorphism were significant lower in patients with CHD history after adjustment for sex and age. The present results suggested that the T allele of rs3745357 in *ARHGEF18* is a protective factor for the initiation of PAH when the patients do not have CHD history. As for the patients who have CHD history, the T allele turned out to be a risk factor for the PAH susceptibility.

In summary, it is biologically plausible that the rs3745357 variant in *ARHGEF18* may have an effect on individual susceptibility to pulmonary hypertension. The effect was contrary to that of the patients with or without CHD history. The results obtained in the study suggest that the *ARHGEF18* rs3745357 variant may be useful as a marker to reflect the genetic susceptibility to niPAH.

## Figures and Tables

**Table 1:** Demographic and clinical characteristics of study subjects.

	Patients	Controls
Sex		
Male	55 (0.47)	98 (0.55)
Female	62 (0.53)	78 (0.44)
Age	74.13 ± 12.55	40.06 ± 12.59
Drink		
Yes	13 (0.11)	
No	104 (0.88)	
Smoke		
Yes	21 (0.17)	
No	96 (0.82)	
Coronary heart disease		
Yes	70 (0.59)	
No	47 (0.40)	
Systolic PAP (mmHg)		
With CHD	60.60 ± 13.35	
Without CHD	60.43 ± 11.57	
Left atrial diameter (mm)		
With CHD	48.88 ± 9.94	
Without CHD	47.37 ± 10.77	
LvIDd (mm)		
With CHD	52.37 ± 8.65	
Without CHD	52.22 ± 11.02	
IVST (mm)		
With CHD	11.02 ± 5.89	
Without CHD	9.95 ± 1.15	
LVEF (%)		
With CHD	59.01 ± 13.14	
Without CHD	56.01 ± 17.85	

LvIDd: left ventricular end diastolic diameter; IVST: interventricular septal thickness; LVEF: left ventricular ejection fraction.

**Table 2:** Allele frequencies of rs3745357 in the ARHGEF18 gene among PAH patients and controls.

	Allele	PAH patients	PAH patients^a^	PAH patients^b^	Controls	*P* value		
		Allele numbers (%)	Allele numbers (%)	Allele numbers (%)	Allele numbers (%)	Overall patients versus controls	PAH^a^ versus controls	PAH^b^ versus controls
Rs3745357	C	106 (45.3)	60 (63.8)	46 (32.9)	157 (45.9)	0.932	**0.001**	**0.017**
	T	128 (54.7)	34 (36.2)	94 (67.1)	195 (54.1)			

^a^PH patients without history of coronary heart disease; ^b^PH patients with history of coronary heart disease.

**Table 3:** Association between the ARHGEF18 rs3745357 polymorphism and risk of pulmonary hypertension without history of coronary heart disease.

Genetic model	Genotype	Patients^a^	Control	Logistic regression		Logistic regression (adjusted)^c^	
		*N* = 47	*N* = 176	OR (95% CI)	*P* value	OR^c^ (95% CI)	*P* value
Codominant	TT	8 (17)	57 (32.4)	1.00		1.00	
	CT	18 (38.3)	81 (46)	1.58 (0.64–3.89)	**0.0053**	1.65 (0.38–7.17)	0.13
	CC	21 (44.7)	38 (21.6)	**3.94 (1.58–9.80)**		4.10 (0.90–18.68)	
Dominant	TT	21 (44.7)	57 (21.5)	1		1.00	
	CT/CC	26 (55.3)	119 (78.5)	**2.34 (1.02–5.32)**	**0.032**	2.48 (0.64–9.60)	0.18
Recessive	TT/CT	39 (83)	138 (70.2)	1.00		1.00	
	CC	8 (17)	38 (29.8)	**2.93 (1.49–5.78)**	**0.0021**	2.90 (0.97–8.73)	0.055
Overdominant	TT/CC	29 (61.7)	62 (51.2)	1.00		1.00	
	CT	18 (38.3)	59 (48.8)	0.73 (0.38–1.41)	0.34	0.67 (0.23–1.94)	0.46

^a^PH patients without history of coronary heart disease; ^c^adjusted by sex and age.

**Table 4:** Association between the ARHGEF18 rs3745357 polymorphism and risk of pulmonary hypertension with history of coronary heart disease.

Genetic model	Genotype	Patients^b^	Control	Logistic regression		Logistic regression (adjusted)^c^	
		*N* = 70	*N* = 176	OR (95% CI)	*P* value	OR^c^ (95% CI)	*P* value
Codominant	TT	37 (52.9)	57 (32.4)	1.00		1.00	
	CT	20 (28.6)	81 (46)	**0.38 (0.20–0.72)**	**0.0092**	0.14 (0.02–1.02)	**0.011**
	CC	13 (18.6)	38 (21.6)	0.53 (0.25–1.12)		**0.03 (0.00–0.46)**	
Dominant	TT	37 (52.9)	57 (32.4)	1		1.00	
	CT/CC	33 (47.1)	119 (67.6)	**0.43 (0.24–0.75)**	**0.0031**	**0.10 (0.02–0.69)**	**0.009**
Recessive	TT/CT	57 (81.4)	138 (78.4)	1.00		1.00	
	CC	13 (18.6)	38 (21.6)	0.83 (0.41–1.67)	0.6	**0.11 (0.01–0.93)**	**0.031**
Overdominant	TT/CC	50 (71.4)	95 (54)	1.00		1.00	
	CT	20 (28.6)	81 (46)	**0.47 (0.26–0.85)**	**0.011**	0.54 (0.12–2.44)	0.42

^b^PH patients with history of coronary heart disease; ^c^adjusted by sex and age.

## Data Availability

The data used to support the findings of this study are available from the corresponding author upon request.
